# The Spine of Two-Particle Fleming–Viot Process in a Bounded Interval

**DOI:** 10.1007/s10959-025-01401-4

**Published:** 2025-02-06

**Authors:** Krzysztof Burdzy, János Engländer, Donald E. Marshall

**Affiliations:** 1 Department of Mathematics, University of Washington, Seattle, WA 98195 USA; 2 Department of Mathematics, University of Colorado, Boulder, CO 80309 USA

**Keywords:** Spine, Fleming–Viot process, Brownian motion, *h*-transform, Laplace equation, Harmonic function, Conformal map, Schwarz–Christoffel formula, 30C20, 30E25, 60J80, 60G17

## Abstract

We show that the spine of the Fleming–Viot process driven by Brownian motion and starting with two particles in a bounded interval has a different law from that of Brownian motion conditioned to stay in the interval forever. Furthermore, we estimate the “extra drift.”

## Introduction

Our objective is to show that the spine of the Fleming–Viot process driven by Brownian motion and starting with two particles in the interval $(0,\pi )$ has a different law from that of a Brownian motion conditioned to stay in the interval forever.

A Fleming–Viot process is a process with a branching structure (but not a branching process according to the terminology adopted in the literature on branching processes). Under very mild assumptions, it has a unique spine, i.e., a trajectory within the branching structure that does not end before the lifetime of the process. When the number of individuals in the population is very large, the distribution of the spine is expected to be very close to the distribution of the driving process conditioned on survival forever; this has been proved in some cases (see the next section for references). There are already examples showing that the distribution of the spine may be different from the distribution of the driving process conditioned on survival forever. We believe that the example analyzed in this paper is more “natural” than the previously published ones.

### Literature Review

The following literature review is partly borrowed from [1]. Fleming–Viot-type processes were originally defined in [2]. In this model, there is a population of fixed size. Every individual moves independently from all other individuals according to the same Markovian transition mechanism, in a domain with a boundary. When an individual hits the boundary, the individual is killed and an individual chosen randomly (uniformly) from the survivors splits into two individuals and the process continues in this manner. The question of whether the process can be continued for all times was addressed in [2–5]. All of these papers studied, among other processes, Fleming–Viot processes driven by Brownian motion.

Every Fleming–Viot process has a unique spine, i.e., a trajectory inside the branching tree that never hits the boundary of the domain where the process is confined; this was proved under strong assumptions in [5, Thm. 4] and later in the full generality in [6].

It was proved in [6] that if the state space is finite and the number of individuals in the population goes to infinity then the distributions of spine processes converge to the distribution of the driving Markov process conditioned on survival forever. The same result has also been proven when the driving process is a diffusion reflected normally off the boundary of a compact domain with soft killing (see [7]), or Brownian motion on a Lipschitz domain with hard killing (see [8]).

In [6], an example was given of a Fleming–Viot process driven by a Markov process on a three-element state space such that one of the elements plays the role of the boundary, the population consists of two individuals, and the distribution of the spine is not equal to the distribution of the driving Markov process conditioned on survival forever. A Markov process with a three-element state space seems to be a rather artificial example in the context of Fleming–Viot models. More recently, it was shown in [1] that the spine of the Fleming–Viot process with two individuals driven by Brownian motions on $[0,\infty )$ has a spine with a distribution different from the distribution of Brownian motion conditioned to stay positive, i.e., the distribution of the 3-dimensional Bessel process. The fact that Brownian motion conditioned to stay positive forever is transient makes that example somewhat special. We hope that the example analyzed in this paper—of a two-particle Fleming–Viot process driven by Brownian motion in $(0,\pi )$—can be considered “completely natural.”

There is a very exciting new development regarding two-particle Fleming–Viot processes, a recent paper [9]. This new paper provides a technique that we use to prove one of our main theorems. It also strengthens motivation for studying this seemingly very specialized stochastic model. The two main results in [9] are the following. First, for a two-particle Fleming–Viot process driven by Brownian motion in a bounded Euclidean domain, the uniform probability measure is stationary for the location of the particles at branching times. Second, the Green function in the domain, considered as a function of two variables, is the stationary density for the Fleming–Viot process. Actually, the results in [9] are much more general—they are concerned with two-particle Fleming–Viot processes driven by any symmetric Markov processes.

### Heuristics

Our objective is to show that the spine of the Fleming–Viot process driven by Brownian motion on $(0,\pi )$ has a different law from that of Brownian motion conditioned to stay in the interval forever.

The basic idea is to represent the two particles moving in $(0,\pi )$ before one of them exits the interval as a single two-dimensional Brownian particle until it hits the boundary of $(0,\pi )^2$. Without loss of generality, we will assume that the vertical component exits $(0,\pi )$ first. Then, until the exit time, the horizontal component represents the spine. We will use the following two facts about conditioned Brownian motion. First, one-dimensional Brownian motion conditioned to never exit $(0,\pi )$ is a space-time Doob’s *H*-process (for an appropriate positive solution *H* of the corresponding heat equation). Second, two-dimensional Brownian motion conditioned to exit $(0,\pi )^2$ via the upper or lower side is a Doob’s *h*-process for an appropriate harmonic function.

#### Remarks on Drift Versus Particle Numbers

Consider *n*-particle Fleming–Viot process driven by Brownian motion in^1^$D:=(0,\pi )$. One might be interested in how the drift of the spine is compared to that of Brownian motion conditioned to stay in the interval forever, for a general particle number $n\ge 2$.

Let $t>0$. A particle (initial ancestor) at time 0 is said to be a “spine candidate at *t*” if not all its descendants have hit $\partial D$ by *t*. Let

1.1 $$\begin{aligned} N_t^{\textsf{cand}}&:=\#\{\text {spine candidates at}\ t \};\end{aligned}$$

1.2 $$\begin{aligned} \tau ^{\textsf{settle}}_n&:=\inf \{s>0\ :\ N_s^{\textsf{cand}}=1\}. \end{aligned}$$

**Fig. 1:**
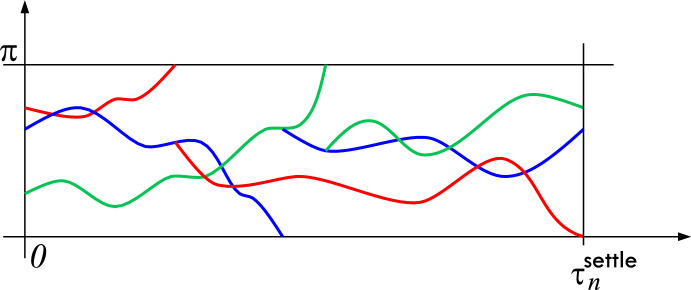
$\tau ^{\textsf{settle}}_n$ is the first time when it is known which of the three particles belongs to the spine at time 0. In this case, it is the green particle. Note that the green particle hits the boundary of the domain before time $\tau ^{\textsf{settle}}_n$

Note that the spine is launched from the unique particle (initial ancestor) with an infinite genealogical tree, and $\tau ^{\textsf{settle}}_n$ is the first time $t>0$ when all genealogical paths for all particles at time *t* have the same root at the time 0. Thus, for $s>\tau ^{\textsf{settle}}_n$, given the Fleming–Viot process’s realization on [0, *s*], the spine trajectory on [0, *t*] can be deduced without ambiguity for all sufficiently small $t's$ (that is, one can determine the “spine stem”); for $s<\tau ^{\textsf{settle}}_n$ this is not possible. See Fig. 1.

In order to develop an intuition about the effect of the particle number on the drift, first consider $n=2$, as in the setup of this paper. In this case, $\tau ^{\textsf{settle}}_2$ agrees with the first hitting time of the boundary by any of the two particles. Indeed, if we start with “blue” and “red” particles, then at the moment the first hit happens by, say, the blue particle, we know that the path of the red particle on any smaller time interval is surely that of the spine. In terms of conditioning, if we want to describe the law of the spine’s trajectory on a time interval [0, *dt*] then the conditioning is not to survive forever, but rather until the other particle hits the boundary. This weaker conditioning results in an inward drift that is weaker than that for Brownian motion conditioned to stay in the interval forever.

Let us also mention in passing that another way to represent the conditioning is by saying that the vector of the two particles has to exit the square $(0,\pi )^2$ via the lower or upper edge. This representation will be the basis of calculations in the rest of the paper but it is harder to translate it into intuition about the strength of the spine drift.

Before turning to $n>2$, let us first note that it is known (see [5, 6]) that $\tau ^{\textsf{settle}}_n<\infty$ almost surely for each fixed $n\ge 2$, but

1.3 $$\begin{aligned} \lim _{n\rightarrow \infty }\tau ^{\textsf{settle}}_n=\infty ,\quad \text {in law}. \end{aligned}$$

For a general *n*, just like for $n=2$ before, we would like to conclude that the spine trajectory on [0, *dt*] (that is the “spine stem”) is launched by the “red” particle. Notice that for $n>2$ the situation becomes more subtle in that it is no longer enough for the red particle to survive until another particle hits the boundary. In fact, recalling the paragraph after (1.1), we see that one has to wait until $\tau ^{\textsf{settle}}_n$ to make such an inference.

This means that for each given $n\ge 2$ one has weaker conditioning than perpetual survival. Namely, the genealogical tree of the initial spine ancestor must survive until the extinction time of the genealogical trees of all other particles; this time is exactly $\tau ^{\textsf{settle}}_n$. Consider now the following cases of conditioning:

1. The conditioning for the tree of the initial ancestor in the previous sentence.
2. Requiring survival for a single Brownian motion starting from the initial ancestor’s position until $\tau ^{\textsf{settle}}_n$.
3. Requiring *perpetual* survival for a single Brownian motion starting at the same position.

Intuitively, the first conditioning is weaker than the second (as a tree has more ways to survive than a single particle), which in turn is clearly weaker than the last one. According to (1.3), in the large particle limit ($n\rightarrow \infty$), the discrepancy between the second and third cases fades away and as it turns out, the condition of the spine stem indeed becomes perpetual survival, in accordance with the main result in [8]. Accordingly, the same can be said about the corresponding (inward) drifts of the spines at time zero.

### On Software Assisted Proofs

As a matter of principle, we tried to provide proofs that are human-verifiable. However, we used Mathematica to obtain certain explicit formulas, exact quantities and approximate quantities. We indicated essential uses of Mathematica in the text.

### Organization of the Paper

The rest of the paper starts with rigorous statements of the model and the three main theorems in Sect. 2. The theorems are proved in the remaining sections. Section 3 is based on Fourier analysis. Next comes Sect. 4 presenting an argument based on complex analysis. Finally, Sect. 5 contains arguments using results from [9], so ultimately it is based on potential theoretic techniques.

## Model and Main Results

We will now define a Fleming–Viot process and other elements of the model. Informally, the process consists of two independent Brownian particles starting at the same point in $(0,\pi )$. At the time when one of them hits 0 or $\pi$, it is killed and the other one branches into two particles. The new particles start moving as independent Brownian motions and the scheme is repeated.

### Notation and Definitions

Let $(W_1(t):t\ge 0)$ and $(W_2(t):t\ge 0)$ be two independent Brownian motions with $W_1(0) =a$, $W_2(0)=b$, $a,b\in (0,\pi )$. Let

$$\begin{aligned} T_0&=0,\\ \tau ^1_j&= \inf \{t\ge 0: W_j(t) =0\text { or }\pi \}, \qquad j=1,2,\\ T_1&=\min (\tau ^1_1,\tau ^1_2),\\ m_1&=j\in \{1 , 2\} \text { such that } \tau ^1_j \ne T_1,\\ Y_1&=W_{m_1}(T_1), \end{aligned}$$

and for $$k\ge 2$$,

$$\begin{aligned} \tau ^k_j&=\inf \{t>T_{k-1} : W_j(t)-W_j(T_{k-1})+Y_{k-1}=0\text { or }\pi \}, \qquad j=1,2,\\ T_k&=\min (\tau ^k_1,\tau ^k_2),\\ m_k&=j \in \{1 , 2\} \text { such that } \tau ^k_j \ne T_k,\\ Y_k&=W_{m_k}(T_k)-W_{m_k}(T_{k-1})+Y_{k-1}. \end{aligned}$$

Note that for a fixed *j*, $\tau ^k_j$ need not be different for different *k*.

It follows from [4, Thm. 5.4] or [5, Thm. 1] that, a.s., $T_k\rightarrow \infty$. Hence, for any $t\ge 0$ we can find $j\ge 1$ such that $t\in [T_{j-1},T_{j})$. Then we set

2.1 $$\begin{aligned} \mathcal {V}(t)&=(V_1(t),V_2(t)) =(W_1(t)-W_1(T_{j-1})+Y_{j-1},W_2(t)-W_2(T_{j-1})+Y_{j-1}). \end{aligned}$$

This completes the definition of $\{\mathcal {V}(t), t\ge 0\}$, an example of a Fleming–Viot process. We will write  and  to denote the probability and expectation associated with $\mathcal {V}$, where $\mu =\delta _{(a,b)}$.

Let $J_t = J(t)$ denote the spine, i.e., $J_t = V_1(t)$ for $t\in [T_{k-1},T_{k})$ if $V_1(T_k-) \in (0,\pi )$. If the last condition fails, we let $J_t = V_2(t)$ for $t\in [T_{k-1},T_{k})$.

Let $\phi$ denote the principal Dirichlet eigenfunction on $(0,\pi )$ for the operator $-\frac{1}{2} \Delta$, i.e., $\phi (x) =\sin x$. The principal eigenvalue is 1/2. The function $H(x,t):= e^{(1/2)t}\phi (x)$ solves the corresponding heat equation. If $W_t$ is one-dimensional Brownian motion and we condition the space-time process $(W_t,t)$ using Doob-conditioning with *H* then we obtain a process $(X_t,t)$ whose first component is “Brownian motion conditioned to stay inside $(0,\pi )$ forever.”

If $a\in (0,\pi )$ and $f\in C_c^2((0,\pi ))$ then2.2Let *h* be the harmonic function in $(0,\pi )^2$ with zero boundary values on the left and right sides and boundary values 1 on the other two sides (see (3.1)). We will write $h_x(x,y) = \frac{\partial }{\partial x}h(x,y)$.

Recall that the harmonic measure is the exit distribution of a Brownian motion, with a given starting (base) point *z*. More precisely, the harmonic measure of a subset of the boundary of a bounded domain *D* in $\mathbb {R}^{2}$ is the probability that a Brownian motion started at $z\in D$ hits that subset of the boundary.

Let $\P ^{BM}_{(a,b)}$ be the distribution of $\{(W_1(t),W_2(t)), 0\le t < T_1\}$ starting from (*a*, *b*) and let $\P ^h_{(a,b)}$ be the distribution of $\{(W_1(t),W_2(t)), 0\le t < T_1\}$ conditioned by $\{\tau ^1_2<\tau ^1_1\}$, i.e., $W_2$ exits from $(0,\pi )$ before $W_1$ does. The distribution $\P ^h_{(a,b)}$ is an example of Doob’s *h*-transform. Note that $h(x,y)=\P ^{BM}_{(x,y)}\left( \tau ^1_2<\tau ^1_1\right)$.

If $a,b\in (0,\pi )$ and $f\in C_c^2((0,\pi ))$ then2.3Note that the drift depends on $W_2$.

### Comparing Generators and Pseudo-Generators

We believe that the spine $J_t$ is not a Markov process relative to its natural filtration $\mathcal {F}^J_t:=\sigma \{J_s: s\le t\}$ (this is why the title of this section contains the term “pseudo-generators”). Nevertheless, one can apply to *J* the same computations that are used for Markov processes and their generators. Our goal is to show that the formula for *J* analogous to (2.2) gives a different result from that in (2.2) so the distributions of $\{X,t\ge 0\}$ and $\{J_t,t\ge 0\}$ must be different.

#### Lemma 2.1

If $a,b\in (0,\pi )$, $\mu =\delta _{(a,b)}$ and $f\in C_c^2((0,\pi ))$ then2.4

#### Proof

First, we will show that2.5For fixed $a,b\in (0,\pi )$,  and  so . This justifies the last equality in the following calculation,Therefore,2.6We have  because if $(a-\epsilon , a+\epsilon )\times (b-\epsilon , b+\epsilon )\subset (0,\pi )^2$ then the probability of exiting $(a-\epsilon , a+\epsilon )\times (b-\epsilon , b+\epsilon )$ before time $t>0$ is exponentially small in 1/*t*. This and  imply that . Since *f* is bounded, these remarks imply that both limits in (2.6) are 0. This completes the proof of (2.5).

In the following computation, the first equality follows from (2.5), the fourth one from a calculation similar to the proof of (2.5), and the last one from (2.4). We complete the proof of the lemma as follows,$\square$

### Main Results

Our main claim is that the law of the spine $\{J_t, 0\le t <\infty \}$ is different from the law of $\{X_t, 0\le t <\infty \}$, i.e., Brownian motion conditioned to stay in $(0,\pi )$ forever. We will state three theorems to this effect. While there is some overlap and redundancy, each theorem and proof supplies extra information not contained in the others. Moreover, the methods of proof of these theorems are totally different thus adding some extra value for the potential future investigations.

In view of (2.2) and Lemma 2.1, it suffices to show that $\phi '/\phi \ne h_x/h$—this is the claim made in the first two theorems.

#### Theorem 2.2

We have

2.7 $$\begin{aligned} \frac{\phi '}{\phi }(\pi /4)- \frac{ h_x}{h}(\pi /4,\pi /4)&> 0, \end{aligned}$$

2.8 $$\begin{aligned} \frac{\phi '}{\phi }(\pi /4)- \frac{ h_x}{h}(\pi /4,\pi /4)&\approx 0.248532, \end{aligned}$$

and consequently, when $a=b=\pi /4$ and $\mu =\delta _{\pi /4,\pi /4}$, *J* under $\textbf{P}_{\mu }$ does not have the same distribution as Brownian motion under $\mathbb {P}^H$ starting at $\pi /4$.

#### Theorem 2.3

For $$0<x,y<\pi /2$$,

$$\begin{aligned} \frac{\phi '}{\phi }(x) >\frac{h_x(x,y)}{h(x,y)}; \end{aligned}$$

by symmetry, analogous inequalities hold in the other parts of $[0,\pi ]^2$. Consequently, if $a,b\in (0,\pi /2)$, $\mu =\delta _{(a,b)}$ and $p=\textbf{P}_{\mu }(J_0=a)$, then *J* under $\textbf{P}_{\mu }$ does not have the same distribution as Brownian motion under $\mathbb {P}^H$ starting at $p\delta _a+(1-p)\delta _b$.

The following result shows the difference between the two laws in a different way.

#### Theorem 2.4

Let $\mathsf {L_1}$ denote the law of the spine $\{J_t, 0\le t <\infty \}$ under  with an arbitrary initial distribution $\mu$ of $\mathcal {V}$. Let $\mathsf {L_2}$ denote the law of $\{X_t, 0\le t <\infty \}$, i.e., Brownian motion conditioned to stay in $(0,\pi )$ forever, with an arbitrary distribution of $X_0$. Then the probability measures $\mathsf {L_1}$ and $\mathsf {L_2}$ on $C([0,\infty ))$ are mutually singular.

We note that the most interesting case covered by Theorem 2.4 is when the distributions of $J_0$ and $X_0$ are identical.

The proof of Theorem 2.4 shows that there exist tail events $A_1$ and $A_2$ such that $\mathsf {L_1}(A_1)=1-\mathsf {L_2}(A_1)=1$ and $1-\mathsf {L_1}(A_2)=\mathsf {L_2}(A_2)=1$.

The proofs of the three theorems will be given in Sects. 3, 4 and 5.

## Drift Comparison at a Branching time at “Midpoint”

We will use Fourier techniques to compare the drifts of the spine at a branching time and Brownian motion conditioned to stay in $(0,\pi )$ forever. In this section, we will limit our calculations to a specific position of the particles at the branching time, namely, $\pi /4$.

### Proof of Theorem 2.2

The function *h* is the unique bounded solution to the boundary value problem

3.1 $$\begin{aligned} {\left\{ \begin{array}{ll} \Delta h=0 \text { on } (0,\pi )\times (0,\pi ),\\ \lim _{x\rightarrow 0}h(x,y)=\lim _{x\rightarrow \pi }h(x,y)=0,\ y\in (0,\pi ),\\ \lim _{y\rightarrow 0}h(x,y)=\lim _{y\rightarrow \pi }h(x,y)=1,\ x\in (0,\pi ).\end{array}\right. } \end{aligned}$$

Existence follows from Theorem 1.3 in [10, page 5]. Uniqueness follows from Lindelöf’s maximum principle, Lemma 1.1 in [10, page 2].

Using the method of separation of variables, the solution has the following infinite series representation (see e.g., Lec. 34 in [11]).

For $n\ge 1$ and $x,y\in (0,1)$, let

$$\begin{aligned} F_n(y)&=\cosh (ny)+\cosh (n(\pi -y)),\\ G_n(y)&=\sinh (ny)+\sinh (n(\pi -y)),\\ a_n&=(2/\pi )\int _0^{\pi }\sin (nx)\, dx=\frac{2}{\pi }\cdot \frac{1-(-1)^n}{n},\\ {\mathcal {X}}_n(x)&=\sin (nx) ,\\ \mathcal {Y}_n(y)&=a_n (F_n(y)-\coth (n\pi )G_n(y)). \end{aligned}$$

Then

$$\begin{aligned} h(x,y)&=\sum _{n=1}^{\infty } {\mathcal {X}}_n(x)\mathcal {Y}_n(y). \end{aligned}$$

Let $\gamma _k:=\coth ((2k+1)\pi )$ and note that $a_{2k}=0$, while $a_{2k+1}=\frac{1}{\pi }\cdot \frac{4}{2k+1}$. We have

3.2 $$\begin{aligned} h(x,y)&=\frac{4}{\pi }\sum _{k=0}^{\infty } \frac{\sin ((2k+1)x)}{2k+1}\left[ F_{2k+1}(y)-\gamma _kG_{2k+1}(y)\right] ,\nonumber \\ h_x(x,y)&=\frac{4}{\pi }\sum _{k=0}^{\infty } \cos ((2k+1)x)\left[ F_{2k+1}(y)-\gamma _kG_{2k+1}(y)\right] ,\nonumber \\ h_x(\pi /4,\pi /4)&=\frac{4}{\pi }\sum _{k=0}^{\infty } \cos ((2k+1)\pi /4)\left[ F_{2k+1}(\pi /4)-\gamma _kG_{2k+1}(\pi /4)\right] . \end{aligned}$$

Note that

3.3 $$\begin{aligned} (\sqrt{2}\cos ((2k+1)\pi /4), k=1,2,\dots ) = (-1,-1,1,1,-1,-1,1,1,-1,-1,1,1, \dots ). \end{aligned}$$

We extend the definitions of $F_n(y)$ and $G_n(y)$ to positive real values of the parameter *n* and we let $c_t:=F_{t}(\pi /4)-\coth (t\pi ) G_{t}(\pi /4)$ for $t>0$.$\square$

### Lemma 3.1

The function $t\rightarrow c_t$ is decreasing.

### Proof

Using Mathematica,

$$\begin{aligned} \frac{dc_t}{dt}&=\frac{\pi }{4}\sinh \left( \frac{\pi t}{4}\right) +\pi {{\,\textrm{csch}\,}}^2\left( \pi t\right) \sinh \left( \frac{\pi t}{4}\right) -\coth \left( \pi t\right) \frac{\pi }{4}\cosh \left( \frac{\pi t}{4}\right) \\  &\qquad +\frac{3\pi }{4}\sinh \left( \frac{3\pi t}{4}\right) +\pi {{\,\textrm{csch}\,}}^2\left( \pi t\right) \sinh \left( \frac{3\pi t}{4}\right) -\coth \left( \pi t\right) \frac{3\pi }{4}\cosh \left( \frac{3\pi t}{4}\right) \\  &=-\frac{1}{4} \pi \sinh \left( \frac{\pi t}{4}\right) \left( \cosh \left( \frac{\pi t}{2}\right) +2\right) \text {sech}^2\left( \frac{\pi t}{2}\right) . \end{aligned}$$

The last expression is negative for $t>0$. $\square$

The lemma and (3.3) imply that for every $j\ge 0$,

3.4 $$\begin{aligned} \sum _{k=1+4j}^{4+4j} \cos ((2k+1)\pi /4)\left[ F_{2k+1}(\pi /4)-\gamma _kG_{2k+1}(\pi /4)\right] \\ = -c_{8j+3}-c_{8j+5}+c_{8j+7}+c_{8j+9} <0.\nonumber \end{aligned}$$

Grouping the terms in (3.2) by four, and starting with $k=1$ (and not $k=0$), and finally, using (3.4), we obtain the estimate

$$\begin{aligned} h_x(\pi /4,\pi /4)&<S:= \frac{4}{\pi } \cos (\pi /4)\left[ F_{1}(\pi /4)-\coth (\pi ) G_{1}(\pi /4)\right] \\&= \frac{2 \sqrt{2}}{\pi }\cosh \left( \frac{\pi }{4}\right) \text {sech}\left( \frac{\pi }{2}\right) \approx 0.475282. \end{aligned}$$

The results on the last line were obtained using Mathematica. Finally, by symmetry, $h(\pi /4,\pi /4)=1/2$, so

$$\begin{aligned} \cot&(\pi /4)- \frac{ h_x}{h}(\pi /4,\pi /4) = 1 - 2 h_x(\pi /4,\pi /4)> 1-2 S\\&=1-2 \frac{2 \sqrt{2}}{\pi }\cosh \left( \frac{\pi }{4}\right) \text {sech}\left( \frac{\pi }{2}\right) \approx 1-2 \cdot 0.475282 = 0.049436 > 0. \end{aligned}$$

This completes the proof of (2.7).

The approximation stated in (2.8) was computed using Mathematica. A partial sum up to $k=5$ in (3.2) gives the accuracy of six significant digits. $\square$

## Analysis of a Harmonic Function Via Conformal Mappings

The Dirichlet problem (3.1) can be transplanted from the square $S:=(0,\pi )\times (0,\pi )$ to any convenient Jordan domain by means of a conformal map. (A bounded region in $\mathbb {C}$ whose boundary is a closed Jordan curve is called a Jordan domain.) Indeed, if $\Omega$ is a Jordan domain, then there is a conformal map *f* of the unit disk *D* onto $\Omega$ which extends to be a homeomorphism of their closures, $\overline{D}$ onto $\overline{\Omega }$, by the Riemann mapping theorem and Carathéodory’s theorem [10, page 13]. For example, if *h* is the solution to (3.1) on the square *S* then $h\circ f$ is the solution on the disk with boundary values 0 and 1 on the (open) arcs of $\partial D$ corresponding to the boundary edges of *S*. Here we use the fact that a harmonic function on *S* is the real part of an analytic function, and hence $h\circ f$ is also harmonic. In this section, we will transfer our problem from the square $(0,\pi )^2$ to the disk. The southwest quarter of the square corresponds to the southwest quadrant of the disk under our map. We examine partial derivatives on the disk instead of the square by the chain rule, but simplify their expressions using a conformal map of the quadrant onto a chosen subset of the right half plane. It is this transformation that allows us to make the somewhat delicate estimates of the partial derivatives.

We will use both complex and real notation—the meaning should be obvious from the context.

The following claim is a special case of the Schwarz–Christoffel formula (see [12], Ch. 6, Sect. 2.2, Exercise 5). Let $D:= \{z\in \mathbb {C}: |z|<1\}$. The analytic function

4.1 $$\begin{aligned} \varphi (z)= \int _0^z \frac{1}{\sqrt{1+w^4}} dw \end{aligned}$$

yields a conformal map of the unit disk *D* onto a square in $\mathbb {C}$ with vertices $\varphi (e^{i\pi /4}), \varphi (e^{i3\pi /4}), \varphi (e^{i5\pi /4}), \varphi (e^{i7\pi /4})$.

We are using the function $z\mapsto \int _0^z \frac{1}{\sqrt{1+w^4}}\, dw$ instead of the more traditional $\int _0^z \frac{1}{\sqrt{1-w^4}}\, dw$ to avoid a rotation.

### Remark 4.1

If $$z=r e^{ik\pi /4}$$ then

$$\begin{aligned} \varphi (z)=\left( \frac{1}{r}\int _0^{r} \frac{1}{\sqrt{1\pm t^4}}\, dt\right) \, z, \end{aligned}$$

where $+$ corresponds to even *k* and − to odd *k*. That is, rays in *D* emanating from 0 at angles that are multiples of $\pi /4$ are mapped by $\varphi$ onto rays in the square with the same slopes.

### Lemma 4.2

Let $c>0$ be such that $c\varphi$ maps the unit disk onto $(-\pi /2,\pi /2)^2$. Then

4.2 $$\begin{aligned}&c= 4\pi ^{3/2}(\Gamma (1/4))^{-2}\approx 1.69443, \end{aligned}$$

where $\Gamma$ is Euler’s Gamma function.

### Proof

We apply Remark 4.1 with $r,k=1$ to see that the diagonal of $(-\pi /2,\pi /2)^2$ is equal to

4.3 $$\begin{aligned} \sqrt{2} \pi = 2c \int _0^{1 } \frac{1}{\sqrt{1- t^4}} dt. \end{aligned}$$

Substituting $u=1-t^4$, it follows that

4.4 $$\begin{aligned} 2c\int _0^{1} \frac{1}{\sqrt{1- t^4}}dt&=\frac{c}{2}\int _0^1 u^{-1/2}(1-u)^{-3/4} du =\frac{c}{2} B(1/2,1/4), \end{aligned}$$

where *B* is the Beta function. By (4.3) and (4.4), using the elementary identities $B(z,w)=\Gamma (z)\Gamma (w)/\Gamma (z+w)$, $\Gamma (w)\Gamma (1-w)=\pi /\sin \pi w$, with $z=\frac{1}{2}$, $w=\frac{1}{4}$, and $\Gamma (\frac{1}{2})=\sqrt{\pi }$ it follows that $c=4\pi ^{3/2}/\Gamma (1/4)^2.$ See p. 46 in [13]. The numerical value of $\Gamma (1/4)$ is well known and can be computed using Gauss’ algorithm for arithmetic–geometric mean (AGM). $\square$

Recall that *h* is the bounded harmonic function in $(0,\pi )^2$ with boundary values 0 on the left and right sides, and 1 on the lower and upper sides.

For any function *f*(*z*) of complex variable $z=x+iy$, we will denote partial derivatives by $f_x,f_y, f_{xy}$, etc.

### Proposition 4.3

For a fixed $0<x<\pi /2$, the function

$$\begin{aligned} y \mapsto \frac{h_x(x+iy)}{h(x+iy)} \end{aligned}$$

is increasing for $0<y<\pi /2$.

### Proof

Let *u* be bounded and harmonic in *D* with boundary values

$$\begin{aligned} u(e^{i\theta })= {\left\{ \begin{array}{ll} 0&  \text {for } -\pi /4< \theta< \pi /4\text { or } 3\pi /4< \theta< 5\pi /4,\\ 1&  \text {for } \pi /4< \theta< 3\pi /4\text { or } 5\pi /4< \theta < 7\pi /4. \end{array}\right. } \end{aligned}$$

We find a formula for *u* as follows. The function $z\rightarrow z^2$ maps *D* onto itself and maps the boundary values of *u* to the following boundary values: 0 for $z\in \partial D$ with $\Re z >0$, and 1 for $z\in \partial D$ with $\Re z <0$. Next, we use the mapping $z \rightarrow (i+z)/(i-z)$ to map *D* onto the right halfplane. Indeed for $z=x+iy,\ x,y\in \mathbb {R},\ x^2+y^2=1$, we have $(i+z)/(i-z)=-i x/(y-1)$. The right half of the circle $\partial D$ is mapped onto the lower half of the vertical axis and the left half of $\partial D$ is mapped onto the upper half of the vertical axis. Hence, the boundary values are mapped onto 0 on the lower half of the vertical axis and 1 on the upper part of the vertical axis. A bounded harmonic function in the right halfplane with these boundary values is $z\rightarrow (1/\pi ) \arg z + 1/2 =\text {Re}(-(i/\pi ) \log z+1/2)$.

It follows that, for $z\in D$,

4.5 $$\begin{aligned} u(z) = \Re \left( -\frac{i}{\pi }\log \left( \frac{i+z^2}{i-z^2}\right) + 1/2\right) . \end{aligned}$$

For $$z\in D$$, let

$$\begin{aligned} f(z) = -\frac{i}{\pi }\log \left( \frac{i+z^2}{i-z^2}\right) + 1/2. \end{aligned}$$

Then

4.6 $$\begin{aligned} f'(z) = -\frac{i}{\pi }\left( \frac{2z}{i+z^2} + \frac{2z}{i-z^2}\right) = - \frac{4}{\pi }\frac{z}{1+z^4}. \end{aligned}$$

By the Cauchy-Riemann equations $f'(z) = f_x(z)=u_x(z) -i u_y(z)$.

Next we map the square $[0,\pi ]^2\subset \mathbb {R}^2=\mathbb {C}$ onto *D* as follows. The mapping $z\mapsto z- \pi (1+i)/2$ maps the square $[0,\pi ]^2$ onto the square with vertices $\pi (1+i)/2, \pi (-1+i)/2, -\pi (1+i)/2, \pi (1-i)/2$. Hence,

4.7 $$\begin{aligned} \psi (z) = \varphi ^{-1} \left( \frac{z- \pi (1+i)/2}{c}\right) \end{aligned}$$

maps $[0,\pi ]^2$ onto *D*, where $c>0$ is defined in Lemma 4.2.

Note that $h = u \circ \psi$ because $u \circ \psi$ is a bounded harmonic function in $[0,\pi ]^2$ with boundary values 0 on the left and right sides, and 1 on the lower and upper sides.

If $w=\psi (z)$ then, using (4.7),

4.8 $$\begin{aligned} \psi ^{-1}(w)&= c \varphi (w) + \pi (1+i)/2, \end{aligned}$$

4.9 $$\begin{aligned} (\psi ^{-1})'(w)&= c \varphi '(w)=\frac{c}{\sqrt{1+w^4}}. \end{aligned}$$

By the Cauchy-Riemann equations, $h_x-ih_y=(f\circ \psi )' = f'\circ \psi ~\psi '$, on the square $S=(0,\pi )^2$. Thus by (4.6) and (4.9), for $z\in D$,

4.10 $$\begin{aligned} (h_x - i h_y)\circ \psi ^{-1}(z)= f'(z)/(\psi ^{-1})'(z)&= \frac{- \frac{4}{\pi }\frac{z}{1+z^4}}{c /\sqrt{1+z^4}} = -\frac{4}{c\pi } \frac{z}{\sqrt{1+z^4}}.\ \end{aligned}$$

We square both sides and compute the imaginary part,

4.11 $$\begin{aligned} -2(h_x\circ \psi ^{-1})(z) (h_y\circ \psi ^{-1})(z)&= \frac{16}{c^2\pi ^2} \Im \left( \frac{z^2}{1+z^4}\right) = \frac{16}{c^2\pi ^2} \Im \left( \frac{1}{z^{-2}+z^2}\right) . \end{aligned}$$

Taking another derivative of (4.10) and using (4.9),

4.12 $$\begin{aligned} ((h_{xx}\circ \psi ^{-1})(z) - i (h_{yx}\circ \psi ^{-1})(z) )(\psi ^{-1})'(z)&= -\frac{4}{c\pi } \frac{1-z^4}{(1+z^4)^{3/2}},\nonumber \\ (h_{xx}\circ \psi ^{-1})(z) - i (h_{yx}\circ \psi ^{-1})(z)&= -\frac{4}{c^2\pi } \frac{1-z^4}{1+z^4}, \end{aligned}$$

4.13 $$\begin{aligned} (h_{yx}\circ \psi ^{-1})(z)&= \frac{4}{c^2\pi } \Im \left( \frac{1-z^4}{1+z^4}\right) \nonumber \\&= \frac{4}{c^2\pi } \Im \left( \frac{z^{-2}-z^2}{z^{-2}+z^2}\right) . \end{aligned}$$

Let $F(z):= h_x(z)/h(z)$ where $z=x+iy$. Then

4.14 $$\begin{aligned} \frac{\partial F}{\partial y} = \frac{h h_{xy} -h_xh_y}{h^2}. \end{aligned}$$

We would like to determine the sign of $F_y$ so it will suffice to analyze the sign of $h h_{xy} -h_xh_y$.

We will change variables as follows,

4.15 $$\begin{aligned} z= \varphi ^{-1}(x+iy), \qquad z^2 = \rho , \qquad w = \frac{i+\rho }{i-\rho }, \qquad w=t e^{i\alpha }, \end{aligned}$$

where $$x+iy\in [0,\pi ]^2$$ and $$z,\rho \in D$$.

We recall from Remark 4.1 that for $0\le r \le 1$ and integer *k*,

$$\begin{aligned} \int _0^{r e^{ik\pi /4}} \frac{1}{\sqrt{1+w^4}}\, dw = e^{ik\pi /4} \int _0^{r } \frac{1}{\sqrt{1\pm t^4}}\, dt, \end{aligned}$$

where $+$ corresponds to even *k* and − to odd *k*. It follows that rays in *D* emanating from 0 at angles that are multiples of $\pi /4$ are mapped by $\varphi$ onto rays in the square with the same slopes. Hence, the regions between rays in *D* are mapped onto the regions between the corresponding rays in the square. We are concerned with the south-west part of the square. It corresponds to *z*-values in the south-west part of *D*. As $z\mapsto z^2$ maps this latter region onto the upper half of D, the corresponding $\rho$-values are in the upper half of *D*.

The function $\rho \mapsto \frac{i+\rho }{i-\rho }$ maps the upper half of *D* onto $\{w\in \mathbb {C}\mid \text {Re}(w)>0, |w|>1\}$, so we must have $t>1$ and $-\pi /2< \alpha < \pi /2$ in (4.15). Using (4.15), we can write $z=z(t,\alpha )$, for $z\in D$. Then

4.16 $$\begin{aligned} h(z(t,\alpha )) = \frac{\alpha }{\pi }+\frac{1}{2}. \end{aligned}$$

We have

$$\begin{aligned}&z^2 = \rho = i \left( \frac{w-1}{w+1}\right) ,\\&z^2 + z^{-2} = i \left( \frac{w-1}{w+1}\right) - i \left( \frac{w+1}{w-1}\right) = i \left( \frac{(w-1)^2 - (w+1)^2}{w^2-1} \right) = -\frac{4iw}{w^2-1},\\&z^2 - z^{-2} = i \left( \frac{(w-1)^2 + (w+1)^2}{w^2-1} \right) =2i \left( \frac{ w^2+1}{w^2-1} \right) ,\\&\Im \left( \frac{z^{-2}-z^2}{z^{-2}+z^2}\right) = \Im \left( \frac{-2i \left( \frac{ w^2+1}{w^2-1}\right) }{ -\frac{4iw}{w^2-1}}\right) = \Im \left( \frac{1}{2} (w + 1/w)\right) \\&= \Im \left( \frac{1}{2} \left( t e^{i\alpha } + \frac{1}{t} e^{-i\alpha }\right) \right) =\frac{1}{2} \left( t - \frac{1}{t}\right) \sin \alpha ,\\&\Im \left( \frac{1}{z^{-2}+z^2}\right) = \Im \left( \frac{i (w^2-1)}{4 w}\right) = \Re \left( \frac{1}{4} \left( w - \frac{1}{w} \right) \right) \\&= \Re \left( \frac{1}{4} \left( t e^{i\alpha } - \frac{1}{t} e^{-i\alpha } \right) \right) = \frac{1}{4} \left( t - \frac{1}{t}\right) \cos \alpha . \end{aligned}$$

It follows immediately from these equations along with (4.16), (4.13) and (4.11) that

$$\begin{aligned}&(( h h_{xy} -h_xh_y)\circ \psi ^{-1})(z(t,\alpha ))\\&\ \ = \left( \frac{\alpha }{\pi }+\frac{1}{2}\right) \frac{4}{c^2\pi }\cdot \frac{1}{2} \left( t - \frac{1}{t}\right) \sin \alpha + \frac{8}{c^2 \pi ^2}\cdot \frac{1}{4} \left( t - \frac{1}{t}\right) \cos \alpha \\&\ \ = \frac{2}{c^2\pi ^2} \left( \left( \alpha + \frac{\pi }{2}\right) \sin \alpha + \cos \alpha \right) \left( t - \frac{1}{t}\right) . \end{aligned}$$

Recall that $t>1$. Hence, $t - \frac{1}{t} >0$. If $g(\alpha ) = \left( \alpha + \frac{\pi }{2}\right) \sin \alpha + \cos \alpha$ then $g(-\pi /2) = 0$ and $g'(\alpha ) = \left( \alpha + \frac{\pi }{2}\right) \cos \alpha >0$ for $-\pi /2< \alpha < \pi /2$. Therefore, $g(\alpha ) \ge 0$ for $-\pi /2\le \alpha \le \pi /2$. We conclude that $(( h h_{xy} -h_xh_y)\circ \psi ^{-1})(x+iy)\ge 0$ for $(x,y)\in [0,\pi /2]^2$ and this implies, in view of (4.14), that $\frac{\partial F}{\partial y} (x+iy)= \frac{\partial (h_x/h)}{\partial y}(x+iy) \ge 0$. $\square$

### Proposition 4.4

For $$z=x+i\pi /2$$, $$0<x<\pi /2$$,

$$\begin{aligned} \frac{h_x(z)}{h(z)} < \cot (\Re z). \end{aligned}$$

### Proof

Let $K(x) = h_x(x+i\pi /2) \sin x - h(x+i\pi /2) \cos x$ for $0\le x \le \pi /2$. It suffices to show that *K* is negative on this interval.

We will analyze the sign of $K_x(x) = (h_{xx} +h)(x)\sin x$. Since $\sin x>0$ for $0< x \le \pi /2$, it will suffice to analyze the sign of $h_{xx}+h$.

Note that $h_x(\pi /2+i\pi /2)=0$ by symmetry. By assumption, $h(i\pi /2)=0$. Hence,

4.17 $$\begin{aligned} K(0)&= h_x(i\pi /2) \cdot 0 - 0\cdot 1=0, \end{aligned}$$

4.18 $$\begin{aligned} K(\pi /2)&= h_x(\pi /2+i\pi /2) \cdot 1 - \frac{1}{2} \cdot 0 = 0. \end{aligned}$$

By (4.12) applied to $z=t+i\cdot 0$,

4.19 $$\begin{aligned} (h_{xx}\circ \psi ^{-1})(z)&= -\frac{4}{c^2\pi } \frac{1-t^4}{1+t^4}, \end{aligned}$$

because the right side of (4.19) is real.

Differentiating (4.12) and applying (4.9) yields

$$\begin{aligned} ((h_{xxx}\circ \psi ^{-1})(z) - i (h_{yxx}\circ \psi ^{-1})(z) )(\psi ^{-1})'(z)&= \frac{4}{c^2\pi } \frac{8z^3}{(1+z^4)^2}, \\ (h_{xxx}\circ \psi ^{-1})(z) - i (h_{yxx}\circ \psi ^{-1})(z)&= \frac{32}{c^3\pi } \frac{z^3}{(1+z^4)^{3/2}}, \\ (h_{xxx}\circ \psi ^{-1})(t)&= \frac{32}{c^3\pi } \frac{t^3}{(1+t^4)^{3/2}}. \end{aligned}$$

The last line follows again by the fact that the right side is real.

By (4.10),

$$\begin{aligned} (h_x\circ \psi ^{-1})(t)&= -\frac{4}{c\pi } \frac{t}{\sqrt{1+t^4}}, \end{aligned}$$

so

$$\begin{aligned} (h_{xxx}\circ \psi ^{-1})(t) + (h_x\circ \psi ^{-1})(t)&=\frac{32}{c^3\pi } \frac{t^3}{(1+t^4)^{3/2}} -\frac{4}{c\pi } \frac{t}{\sqrt{1+t^4}}\\&= \frac{4}{c\pi } \frac{(-t)}{(1+t^4)^{3/2}} \left( t^4-\frac{8}{c^2} t^2 + 1 \right) . \end{aligned}$$

We want to determine the sign of the last expression so it will suffice to analyze $$p(t):=t^4-( 8/ {c^2}) t^2 + 1 $$. The roots of this polynomial must satisfy

$$\begin{aligned} t^2 = \frac{1}{2} \left( \frac{8}{c^2} \pm \sqrt{64/c^4 -4} \right) = \frac{4}{c^2} \pm \sqrt{\left( \frac{4}{c^2}\right) ^2 -1}. \end{aligned}$$

Since $$c< 2 $$ (see Lemma 4.2), these values of $$t^2$$ are positive and distinct. Moreover, their product is 1. Hence, in $$(-1,0)$$, the function *p* has a single root $$t_0$$.

Note that

$$\begin{aligned} (h_{xxx}\circ \psi ^{-1})(0) + (h_x\circ \psi ^{-1})(0)&= 0,\\ (h_{xxx}\circ \psi ^{-1})(-1) + (h_x\circ \psi ^{-1})(-1)&= \frac{4}{c\pi } \frac{-1}{(1+1)^{3/2}} \left( \frac{8}{c^2} - 1 - 1\right) <0. \end{aligned}$$

The last two equations along with the fact that *p* has exactly one root at $t_0$ on $(-1,0)$ imply that for some $0< x_0 < \pi /2$,

$$\begin{aligned}&((h_{xx} +h)_x\circ \psi ^{-1})(t) {\left\{ \begin{array}{ll}<0 &  \text { for } -1<t<t_0,\\>0 &  \text { for } t_0<t<0, \end{array}\right. }\\&(h_{xx} +h)_x(x) {\left\{ \begin{array}{ll}<0 &  \text { for } 0<x<x_0,\\ >0 &  \text { for } x_0<x<\pi /2. \end{array}\right. } \end{aligned}$$

By (3.1) and (4.19),

$$\begin{aligned} ((h_{xx}+h)\circ \psi ^{-1})(-1)&= -\frac{4}{c^2\pi } \frac{1-1}{1+1}+0=0,\\ ((h_{xx}+h)\circ \psi ^{-1})(0)&= -\frac{4}{c^2\pi }+ \frac{1}{2} >0. \end{aligned}$$

These observations imply that the function $(h_{xx}+h)(x)$ decreases from 0 to a minimum at $x_0$ and then increases to a positive maximum at $\pi /2$. Thus for some $0< x_1< \pi /2$, $K_x(x)=(h_{xx}+h)(x)\sin x$ is negative for $0<x< x_1$ and positive for $x_1< x< \pi /2$. In view of (4.17)-(4.18), *K* is negative on the interval $[0,\pi /2]$. $\square$

### Proof (Proof of Theorem 2.3)

The theorem follows from Propositions 4.3 and 4.4. $\square$

## Estimates Based on the Stationary Distribution

We will show that for sufficiently small $\varepsilon >0$, for some $c_{\varepsilon }<C_{\varepsilon }$, the long-term occupation measure of $(0, \varepsilon )$ for Brownian motion conditioned to stay inside $(0,\pi )$ is smaller than $c_\varepsilon$ while the long-term occupation measure of $(0, \varepsilon )$ for the spine is larger than $C_\varepsilon$.

We start with an elementary result based on well-known estimates of harmonic measure and complex analytic methods.

Recall that the harmonic measure is the exit distribution of a Brownian motion, with a given starting (base) point *z*. More precisely, the harmonic measure of a subset of the boundary of a bounded domain *D* in $\mathbb {R}^{2}$ is the probability that a Brownian motion started at $z\in D$ hits that subset of the boundary. For a domain *D*, $A\subset \partial D$ and $z\in D$, let $\nu _z(A)$ denote the harmonic measure of *A* in *D* with the base point *z*.

### Lemma 5.1

(i) If $D= \{v\in \mathbb {C}: |\Im v - a|< r, \Re v < b\}$, $A=\{v\in \partial D: \Re v = b\}$ and $z=c+ ai$ where $c< b$, then

$$\begin{aligned} \nu _z(A)= \frac{4}{\pi }\arctan \left( \exp \left( -\frac{\pi (b-c)}{2r}\right) \right) . \end{aligned}$$

(ii) If $D = \{v\in \mathbb {C}: \Re v>0, \Im v > 0, |v| < \pi \}$, $A= \{v\in \partial D: |v| = \pi \}$ and $z=x+yi\in D$, then

$$\begin{aligned} \nu _z(A) \le \frac{4}{\pi }\arctan ((x^2+y^2)/\pi ^2). \end{aligned}$$

### Proof

(i) Our formula is obtained by scaling from the formula stated on the line after (5.3) on page 144 in [10].

(ii) If $x=y$, we can transform the problem to that in part (i) by using the conformal mapping $z\mapsto \log z$ and then applying conformal invariance of harmonic measure (use $a=\pi /4, b=\log \pi , c=\log (\sqrt{2}x),r=\pi /4$). In the case $x=y$, the formula holds with the equality sign. To extend the formula to the case $x\ne y$, we note that the function $z\mapsto \nu _z(A)$ takes the highest value in the middle of the arc $\{z\in D: |z|^2 =x^2+y^2\}$. The last remark follows from (5.3) on page 144 in [10]. $\square$

### Proof of Theorem 2.4

Since the normalized principal Dirichlet eigenfunction of the Laplacian in $(0,\pi )$ is $\phi (x)=\sqrt{2/\pi }\sin (x)$, the stationary density for Brownian motion $X_t$ conditioned to stay in $(0,\pi )$ forever is $\phi ^2(x)$=$(2/\pi ) \sin ^2 x$. Hence, by the ergodic theorem, a.s.,

5.1 $$\begin{aligned}&\lim _{t\rightarrow \infty } \frac{1}{t} \int _0^t \textbf{1}_{(0,\varepsilon )}(X_s) ds = \int _0^\varepsilon (2/\pi ) \sin ^2 x dx = \frac{1}{\pi }(\varepsilon - (1/2) \sin (2\varepsilon ))\nonumber \\&= \frac{2 \varepsilon ^3}{3 \pi }-\frac{2 \varepsilon ^5}{15 \pi }+o\left( \varepsilon ^6\right) . \end{aligned}$$

Let *G*(*x*, *y*) denote the Green function for Brownian motion in $(0,\pi )$ killed upon exiting the interval. According to [9, Thm. 1.5], *G*(*x*, *y*), appropriately normalized, is the stationary density for the two-particle Fleming–Viot process in $(0,\pi )$ driven by Brownian motion.

We have

$$\begin{aligned} G(x,y) = {\left\{ \begin{array}{ll} \frac{2}{\pi ^2} y (\pi -x) &  \text { if } 0<y< x< \pi ,\\ \frac{2}{\pi ^2} x (\pi - y) &  \text { if } 0<x< y< \pi . \end{array}\right. } \end{aligned}$$

The normalizing constant $\frac{2}{\pi ^2}$ is chosen so that $\int _0^\pi \int _0^\pi G(x,y) dx dy=1$.

Suppose that the process $\mathcal {V}(t)=(V_1(t),V_2(t))$ is in the stationary regime. Consider $x,y\in (0, \pi /2)$. At any time $t\ge 0$, the probability that the spine is in *dx* and it is $V_1(t)$, and $V_2(t)\in dy$, is equal to *G*(*x*, *y*)*dxdy* times the probability *p*(*x*, *y*) that $V_2$ will exit $(0,\pi )$ before $V_1$ does.

Suppose that $y \le x^{1/2}$. The probability *p*(*x*, *y*) is bounded below by the probability $p_1$ that two-dimensional Brownian motion starting from (*x*, *y*) will hit the *x*-axis before it hits the *y*-axis minus the probability $p_2$ that $(V_1,V_2)$ will hit the circle centered at 0 with radius $\pi$ before $(V_1,V_2)$ exits the first quadrant. We have $p_1=(2/\pi )\arctan (x/y)$ because this is the unique bounded harmonic function in the first quadrant with boundary values 0 on the vertical axis and 1 on the horizontal axis. By Lemma 5.1 (ii),

$$\begin{aligned} p_2\le \frac{4}{\pi }\arctan ((x^2+y^2)/\pi ^2). \end{aligned}$$

Hence, in this case,

5.2 $$\begin{aligned} p(x,y)&\ge p_1-p_2 \ge \frac{2}{\pi }\arctan (x/y) - \frac{4}{\pi }\arctan ((x^2+y^2)/\pi ^2). \end{aligned}$$

For sufficiently small $x>0$, since we are assuming that $y \le x^{1/2}$,

$$\begin{aligned} \frac{1}{2} \cdot \frac{2}{\pi }\arctan (x/y) \ge \frac{1}{2} \cdot \frac{2}{\pi }\arctan (x^{1/2})&\ge \frac{4}{\pi }\arctan ((x^2+x)/\pi ^2)\\&\ge \frac{4}{\pi }\arctan ((x^2+y^2)/\pi ^2). \end{aligned}$$

This and (5.2) imply that for sufficiently small $x>0$,

$$\begin{aligned} p(x,y)&\ge \frac{1}{\pi }\arctan (x/y) . \end{aligned}$$

Factor 2 in the following formula is to account for both $V_1$ and $V_2$ possibly being the spine. In the calculation below we will use the estimates $\arctan (x/y) \ge (\pi /4) x/y$ for $y\ge x$, and $\pi -y > \pi -1$, for $y\in (0,1)$. The two-particle process $\mathcal {V}(t)$ in (2.1) is ergodic by [9, Thm. 1.5]. It follows from the ergodic theorem that for sufficiently small $\varepsilon \in (0,1)$, a.s.,

$$\begin{aligned} \lim _{t\rightarrow \infty }&\frac{1}{t} \int _0^t \textbf{1}_{(0,\varepsilon )}(J_s) ds =\int _0^\varepsilon 2\int _0^1 G(x,y) p(x,y) d y dx\\&\ge \int _0^\varepsilon 2\int _x^{x^{1/2}} \frac{2}{\pi ^2} x (\pi -y) \frac{1}{\pi }\arctan (x/y)dy dx\\&\ge c_1 \int _0^\varepsilon \int _x^{x^{1/2}} \frac{x^2}{y}dy dx =\frac{c_1}{6} \varepsilon ^3\left( \log \frac{1}{\varepsilon }+\frac{1}{3}\right) . \end{aligned}$$

This is larger than the quantity in (5.1) for small $\varepsilon >0$. It follows that the distribution of the spine and the distribution of Brownian motion conditioned to stay in $(0,\pi )$ forever are mutually singular. $\square$

## Data Availability

All data generated or analyzed during this study are included in this article.

## Footnotes

1 In fact, the argument below works for any nice bounded Euclidean domain $D\subset {\mathbb {R}}^d,d\ge 1$
